# 4-(Dimethyl­amino)pyridinium 2-(4-hydroxy­phenyl­diazen­yl)benzoate

**DOI:** 10.1107/S1600536809050132

**Published:** 2009-11-28

**Authors:** Hadi D. Arman, Tyler Miller, Edward R. T. Tiekink

**Affiliations:** a Department of Chemistry, The University of Texas at San Antonio, One UTSA Circle, San Antonio, Texas 78249-0698, USA; b Department of Chemistry, University of Malaya, 50603 Kuala Lumpur, Malaysia

## Abstract

In the title molecular salt, C_7_H_11_N_2_^+^·C_13_H_9_N_2_O_3_^−^, the dihedral angle between the benzene rings in the anion is 35.14 (8)°. In the crystal, centrosymmetrically related anions associate *via* hydrox­yl–carboxyl­ate O—H⋯O hydrogen bonds, resulting in a 24-membered {⋯OC_3_N_2_C_4_OH}_2_ synthon. The cations are associated with this dimeric unit *via* pyridinium–carboxyl­ate N—H⋯O hydrogen bonds. Weak C—H⋯O links further consolidate the packing, generating layers.

## Related literature

For a discussion of co-crystal terminology, see: Zukerman-Schpector & Tiekink (2008 ▶). For related co-crystallization studies, see: Broker & Tiekink (2007 ▶); Broker *et al.* (2008 ▶); Ellis *et al.* (2009 ▶). For related investigations with 2-(4-hydroxy­phenyl­azo)benzoic acid, see: Corlette & Tiekink (2009 ▶); Arman *et al.* (2009 ▶). For hydrogen-bond motifs, see: Etter (1990 ▶).
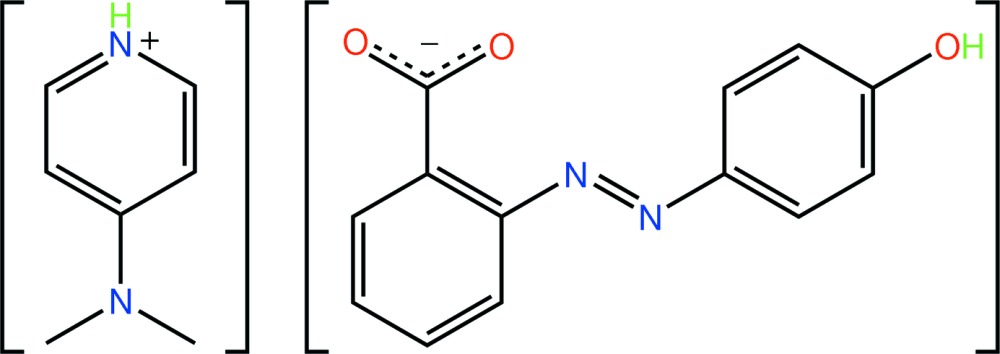


## Experimental

### 

#### Crystal data

C_7_H_11_N_2_^+^·C_13_H_9_N_2_O_3_^−^
*M* *_r_* = 364.40
Monoclinic, 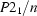

*a* = 9.240 (4) Å
*b* = 10.924 (4) Å
*c* = 17.598 (7) Å
β = 92.002 (8)°
*V* = 1775.2 (12) Å^3^
*Z* = 4
Mo *K*α radiation
μ = 0.09 mm^−1^
*T* = 98 K
0.35 × 0.25 × 0.15 mm

#### Data collection

Rigaku AFC12K/SATURN724 diffractometer
Absorption correction: multi-scan (*ABSCOR*; Higashi, 1995 ▶) *T*_min_ = 0.769, *T*_max_ = 1
12887 measured reflections
4060 independent reflections
3553 reflections with *I* > 2σ(*I*)
*R*_int_ = 0.046

#### Refinement

*R*[*F* ^2^ > 2σ(*F* ^2^)] = 0.055
*wR*(*F* ^2^) = 0.135
*S* = 1.12
4060 reflections
252 parameters
2 restraints
H-atom parameters constrained
Δρ_max_ = 0.33 e Å^−3^
Δρ_min_ = −0.32 e Å^−3^

### 

Data collection: *CrystalClear* (Rigaku/MSC, 2005 ▶); cell refinement: *CrystalClear*; data reduction: *CrystalClear*; program(s) used to solve structure: *SHELXS97* (Sheldrick, 2008 ▶); program(s) used to refine structure: *SHELXL97* (Sheldrick, 2008 ▶); molecular graphics: *ORTEPII* (Johnson, 1976 ▶) and *DIAMOND* (Brandenburg, 2006 ▶); software used to prepare material for publication: *publCIF* (Westrip, 2009 ▶).

## Supplementary Material

Crystal structure: contains datablocks global, I. DOI: 10.1107/S1600536809050132/hb5243sup1.cif

Structure factors: contains datablocks I. DOI: 10.1107/S1600536809050132/hb5243Isup2.hkl

Additional supplementary materials:  crystallographic information; 3D view; checkCIF report

## Figures and Tables

**Table 1:** Hydrogen-bond geometry (Å, °)

*D*—H⋯*A*	*D*—H	H⋯*A*	*D*⋯*A*	*D*—H⋯*A*
O3—H3o⋯O2^i^	0.84	1.77	2.602 (2)	174
N3—H3n⋯O1	0.88	1.78	2.641 (2)	166
C15—H15⋯O2^ii^	0.95	2.41	3.300 (3)	157
C19—H19a⋯O3^iii^	0.98	2.58	2.968 (3)	103

Symmetry codes: (i) 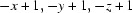; (ii) 
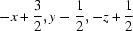
; (iii) 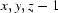.

**Fig. 1.**
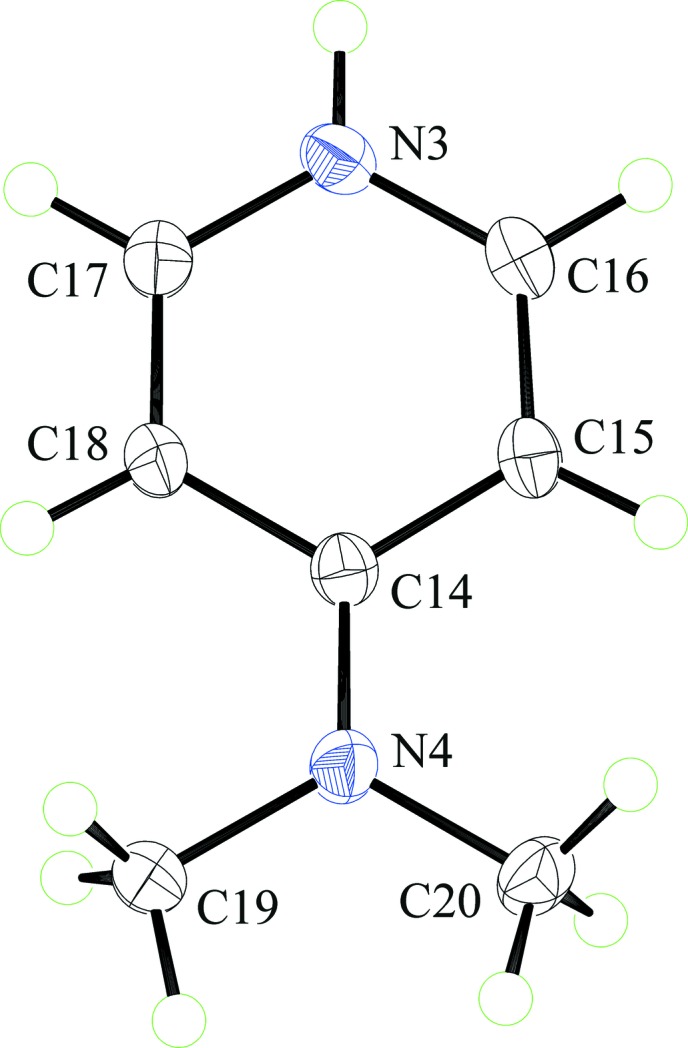
Molecular structure of the p-dimethylaminopyridinium cation in (I), showing displacement ellipsoids at the 50% probability level.

**Fig. 2.**
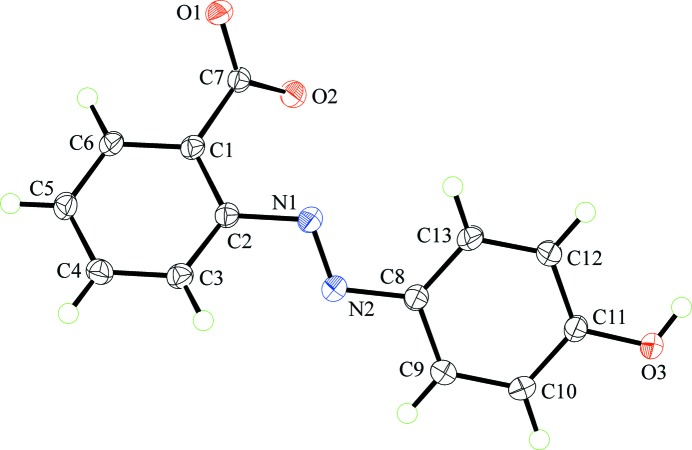
Molecular structure of the 2-(4-hydroxyphenylazo)benzoate anion in (I), showing displacement ellipsoids at the 50% probability level.

**Fig. 3.**
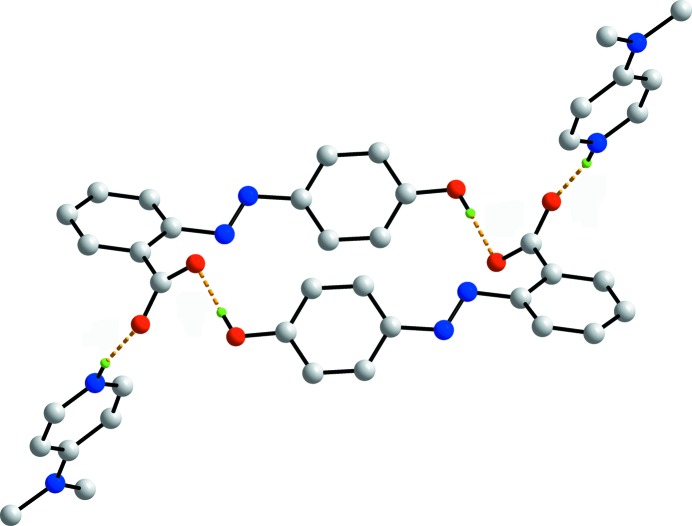
A view of a supramolecular dimer mediated by O—H···O hydrogen bonds (orange dashed lines) highlighting the centrosymmetric 24-membered {···OC3N2C4OH}2 synthon formed between anions, and the attachment of two cations via N—H···O hydrogen bonding (orange dashed lines). Hydrogen atoms not participating in hydrogen bonding are omitted for clarity. Colour code: O, red; N, blue; C, grey; and H, green.

**Fig. 4.**
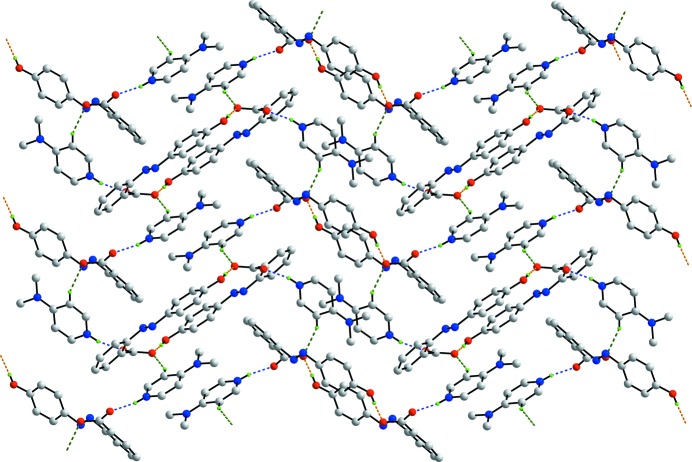
A view of the layer in (I) in which the aggregates shown in Fig. 3 are linked via C–H···O contacts (green dashed lines). Hydrogen atoms not participating in intermolecular interactions are omitted for clarity. Colour code: O, red; N, blue; C, grey; and H, green.

## supplementary crystallographic information

### Comment

The motivation of our studies into co-crystal formation (Broker & Tiekink, 2007; Broker *et al.*, 2008; Ellis *et al.*, 2009) revolves around a desire to establish a hierarchy of hydrogen bonding interactions (Etter, 1990). Studies with 2-(4-hydroxyphenylazo)benzoic acid reveal the formation of a co-crystal when co-crystallized with *N*,*N'*-bis(4-pyridylmethyl)oxamide (Arman *et al.*, 2009) but a salt was formed with 1,2-(4-pyridyl)ethane (Corlette & Tiekink, 2009); see Zukerman-Schpector & Tiekink (2008) for a discussion on terminology.

In (I), proton transfer from the carboxylic acid group in 2-(4-hydroxyphenylazo)benzoic acid to the pyridine-N1 atom has occurred during co-crystallization from a methanol solution containing stoichiometric amounts of the reagents, Figs 1 and 2. The NMe_2_ group in the cation is co-planar with the (N3, C14—C18) ring as seen in the C15–C14–N4–C19 torsion angle of -179.04 (17) °. By contrast, the 2-(4-hydroxyphenylazo)benzoate anion is non-planar with the dihedral angle formed between the (C1—C6) and (C8—C13) rings being 35.14 (8) °. There is a twist about the C2–N1 bond as seen in the C1–C2–N1–N2 torsion angle of -148.88 (15) °. Further, the carboxylate group is twisted out of the benzene ring to which it is connected: the C2–C1–C7–O1 torsion angle is -134.83 (17) °. The C7–O1 and C7–O2 bond distances of 1.259 (2) Å and 1.260 (2) Å are indistinguishable, consistent with deprotonation.

The crystal packing in (I) is dominated by O–H···O and N–H···O hydrogen bonding. Centrosymmetrically related anions associate by hydrogen bonds formed between the hydroxyl and O2-carboxylate groups. The association leads to the formation of a 24-membered {···OC_3_N_2_C_4_OH}_2_ synthon, Fig. 3. Two cations associate with the dimeric aggregate *via* pyridinium-*N*···O1-carboxylate hydrogen bonds, Fig. 3. The resulting four component tectons are linked into effectively flat 2-D arrays in the (100) plane *via* C–H···O contacts, Table 1 and Fig. 4. Links between layers comprise weaker C–H···O contacts, Table 1, and C–H···π interactions: C20–H20*a*···*Cg*(N3,C14—C18)^i^ = 2.61 Å, C20···*Cg*(N2, C14—C18)^i^ = 3.527 (3) Å, with an angle subtended at H20*a* = 155 °; i: 2 - *x*, 1 - *y*, -*z*.

### Experimental

Orange blocks of (I) were isolated from the co-crystallization of 1:1 molar equivalents of 2-(4-hydroxyphenylazo)benzoic acid and *p*-dimethylpyridine in a methanol solution.

### Refinement

C-bound H-atoms were placed in calculated positions (C–H 0.95–0.98 Å) and were included in the refinement in the riding model approximation with *U*_iso_(H) set to 1.2–1.5*U*_eq_(C). A rotating group model was used for the methyl groups. The O– and N– bound H-atoms were located in a difference Fourier map and refined with O–H and N—H restraints of 0.840±0.001 Å and 0.88±0.001, respectively, and with *U*_iso_(H) = n*U*_eq_(carrier atom); n = 1.5 for carrier atom = O, and 1.2 for N.

### Crystal data

**Table:**

C_7_H_11_N_2_^+^·C_13_H_9_N_2_O_3_^−^	*F*(000) = 768
*M**_r_* = 364.40	*D*_x_ = 1.363 Mg m^−^^3^
Monoclinic, *P*2_1_/*n*	Mo *K*α radiation, λ = 0.71069 Å
Hall symbol: -P 2yn	Cell parameters from 7530 reflections
*a* = 9.240 (4) Å	θ = 2.2–40.7°
*b* = 10.924 (4) Å	µ = 0.09 mm^−^^1^
*c* = 17.598 (7) Å	*T* = 98 K
β = 92.002 (8)°	Block, orange
*V* = 1775.2 (12) Å^3^	0.35 × 0.25 × 0.15 mm
*Z* = 4	

### Data collection

**Table:**

Rigaku AFC12K/SATURN724 diffractometer	4060 independent reflections
Radiation source: fine-focus sealed tube	3553 reflections with *I* > 2σ(*I*)
graphite	*R*_int_ = 0.046
ω scans	θ_max_ = 27.5°, θ_min_ = 2.2°
Absorption correction: multi-scan (*ABSCOR*; Higashi, 1995)	*h* = −11→12
*T*_min_ = 0.769, *T*_max_ = 1	*k* = −14→14
12887 measured reflections	*l* = −20→22

### Refinement

**Table:**

Refinement on *F*^2^	Primary atom site location: structure-invariant direct methods
Least-squares matrix: full	Secondary atom site location: difference Fourier map
*R*[*F*^2^ > 2σ(*F*^2^)] = 0.055	Hydrogen site location: inferred from neighbouring sites
*wR*(*F*^2^) = 0.135	H-atom parameters constrained
*S* = 1.12	*w* = 1/[σ^2^(*F*_o_^2^) + (0.0531*P*)^2^ + 0.902*P*] where *P* = (*F*_o_^2^ + 2*F*_c_^2^)/3
4060 reflections	(Δ/σ)_max_ < 0.001
252 parameters	Δρ_max_ = 0.33 e Å^−^^3^
2 restraints	Δρ_min_ = −0.32 e Å^−^^3^

### Special details

**Table:**

Geometry. All e.s.d.'s (except the e.s.d. in the dihedral angle between two l.s. planes) are estimated using the full covariance matrix. The cell e.s.d.'s are taken into account individually in the estimation of e.s.d.'s in distances, angles and torsion angles; correlations between e.s.d.'s in cell parameters are only used when they are defined by crystal symmetry. An approximate (isotropic) treatment of cell e.s.d.'s is used for estimating e.s.d.'s involving l.s. planes.
Refinement. Refinement of *F*^2^ against ALL reflections. The weighted *R*-factor *wR* and goodness of fit *S* are based on *F*^2^, conventional *R*-factors *R* are based on *F*, with *F* set to zero for negative *F*^2^. The threshold expression of *F*^2^ > σ(*F*^2^) is used only for calculating *R*-factors(gt) *etc*. and is not relevant to the choice of reflections for refinement. *R*-factors based on *F*^2^ are statistically about twice as large as those based on *F*, and *R*- factors based on ALL data will be even larger.

### Fractional atomic coordinates and isotropic or equivalent isotropic displacement parameters (Å^2^)

**Table:**

	*x*	*y*	*z*	*U*_iso_*/*U*_eq_	
O1	0.52078 (13)	0.70075 (11)	0.20136 (6)	0.0231 (3)	
O2	0.51014 (13)	0.74136 (12)	0.32532 (6)	0.0248 (3)	
O3	0.72197 (13)	0.39214 (12)	0.69176 (7)	0.0252 (3)	
H3O	0.6507	0.3446	0.6871	0.038*	
N1	0.79272 (15)	0.65156 (12)	0.37743 (8)	0.0198 (3)	
N2	0.86170 (15)	0.66758 (13)	0.43984 (8)	0.0213 (3)	
N3	0.65513 (17)	0.60354 (14)	0.08591 (8)	0.0244 (3)	
H3N	0.6072	0.6460	0.1193	0.029*	
N4	0.86335 (17)	0.38316 (14)	−0.06666 (8)	0.0253 (3)	
C1	0.73363 (18)	0.77410 (15)	0.26482 (9)	0.0186 (3)	
C2	0.83605 (18)	0.73634 (14)	0.32047 (9)	0.0187 (3)	
C3	0.97934 (18)	0.77841 (15)	0.31800 (9)	0.0210 (3)	
H3	1.0502	0.7485	0.3537	0.025*	
C4	1.01826 (19)	0.86322 (16)	0.26387 (9)	0.0229 (4)	
H4	1.1151	0.8924	0.2631	0.028*	
C5	0.91532 (19)	0.90559 (16)	0.21068 (9)	0.0228 (4)	
H5	0.9406	0.9662	0.1748	0.027*	
C6	0.77532 (18)	0.85902 (15)	0.21011 (9)	0.0206 (3)	
H6	0.7068	0.8852	0.1720	0.025*	
C7	0.57669 (18)	0.73456 (14)	0.26416 (9)	0.0183 (3)	
C8	0.82313 (18)	0.58820 (15)	0.49950 (9)	0.0200 (3)	
C9	0.8914 (2)	0.61107 (19)	0.56988 (10)	0.0318 (4)	
H9	0.9628	0.6736	0.5741	0.038*	
C10	0.8577 (2)	0.5449 (2)	0.63363 (10)	0.0331 (5)	
H10	0.9058	0.5618	0.6811	0.040*	
C11	0.75306 (18)	0.45340 (15)	0.62834 (9)	0.0204 (3)	
C12	0.6840 (2)	0.42969 (16)	0.55792 (10)	0.0248 (4)	
H12	0.6124	0.3674	0.5538	0.030*	
C13	0.7188 (2)	0.49608 (16)	0.49430 (9)	0.0244 (4)	
H13	0.6715	0.4789	0.4467	0.029*	
C14	0.79702 (18)	0.45414 (15)	−0.01652 (9)	0.0213 (3)	
C15	0.82162 (19)	0.44292 (16)	0.06333 (9)	0.0230 (3)	
H15	0.8879	0.3836	0.0832	0.028*	
C16	0.7501 (2)	0.51729 (16)	0.11111 (10)	0.0249 (4)	
H16	0.7675	0.5084	0.1644	0.030*	
C17	0.6284 (2)	0.61699 (17)	0.01041 (10)	0.0261 (4)	
H17	0.5608	0.6771	−0.0071	0.031*	
C18	0.6963 (2)	0.54610 (17)	−0.04107 (10)	0.0255 (4)	
H18	0.6764	0.5580	−0.0939	0.031*	
C19	0.8367 (2)	0.39889 (19)	−0.14862 (10)	0.0307 (4)	
H19A	0.7346	0.3821	−0.1615	0.046*	
H19B	0.8978	0.3419	−0.1762	0.046*	
H19C	0.8599	0.4831	−0.1630	0.046*	
C20	0.9618 (2)	0.28541 (17)	−0.04192 (11)	0.0289 (4)	
H20A	1.0554	0.3207	−0.0258	0.043*	
H20B	0.9755	0.2285	−0.0841	0.043*	
H20C	0.9207	0.2414	0.0008	0.043*	

### Atomic displacement parameters (Å^2^)

**Table:**

	*U*^11^	*U*^22^	*U*^33^	*U*^12^	*U*^13^	*U*^23^
O1	0.0229 (6)	0.0285 (6)	0.0177 (5)	−0.0021 (5)	−0.0007 (4)	−0.0009 (5)
O2	0.0268 (6)	0.0295 (7)	0.0182 (6)	−0.0064 (5)	0.0033 (5)	−0.0022 (5)
O3	0.0237 (6)	0.0323 (7)	0.0196 (6)	−0.0071 (5)	−0.0010 (5)	0.0071 (5)
N1	0.0228 (7)	0.0189 (7)	0.0177 (6)	0.0007 (5)	0.0005 (5)	0.0003 (5)
N2	0.0226 (7)	0.0223 (7)	0.0189 (7)	−0.0005 (5)	−0.0002 (5)	0.0014 (6)
N3	0.0289 (8)	0.0249 (7)	0.0194 (7)	−0.0001 (6)	0.0029 (6)	−0.0015 (6)
N4	0.0280 (8)	0.0263 (8)	0.0214 (7)	0.0064 (6)	−0.0031 (6)	−0.0024 (6)
C1	0.0226 (8)	0.0186 (7)	0.0145 (7)	−0.0009 (6)	0.0008 (6)	−0.0018 (6)
C2	0.0233 (8)	0.0170 (7)	0.0157 (7)	−0.0012 (6)	0.0013 (6)	−0.0017 (6)
C3	0.0224 (8)	0.0226 (8)	0.0181 (7)	−0.0001 (6)	0.0002 (6)	−0.0014 (6)
C4	0.0222 (8)	0.0256 (8)	0.0210 (8)	−0.0043 (7)	0.0017 (6)	−0.0031 (7)
C5	0.0288 (9)	0.0234 (8)	0.0163 (7)	−0.0045 (7)	0.0030 (6)	0.0005 (6)
C6	0.0251 (8)	0.0219 (8)	0.0146 (7)	−0.0021 (6)	−0.0009 (6)	0.0003 (6)
C7	0.0213 (8)	0.0167 (7)	0.0167 (7)	−0.0001 (6)	0.0006 (6)	0.0014 (6)
C8	0.0216 (8)	0.0202 (8)	0.0183 (8)	0.0001 (6)	0.0007 (6)	0.0013 (6)
C9	0.0311 (10)	0.0398 (11)	0.0241 (9)	−0.0168 (8)	−0.0049 (7)	0.0059 (8)
C10	0.0337 (10)	0.0447 (11)	0.0204 (8)	−0.0158 (9)	−0.0081 (7)	0.0069 (8)
C11	0.0204 (8)	0.0226 (8)	0.0182 (7)	0.0010 (6)	0.0013 (6)	0.0037 (6)
C12	0.0306 (9)	0.0229 (8)	0.0208 (8)	−0.0084 (7)	−0.0021 (7)	0.0005 (7)
C13	0.0314 (9)	0.0244 (8)	0.0171 (8)	−0.0051 (7)	−0.0037 (7)	0.0001 (7)
C14	0.0232 (8)	0.0193 (8)	0.0211 (8)	−0.0010 (6)	−0.0005 (6)	0.0011 (6)
C15	0.0262 (9)	0.0216 (8)	0.0211 (8)	−0.0017 (6)	−0.0015 (6)	0.0049 (7)
C16	0.0299 (9)	0.0256 (8)	0.0192 (8)	−0.0036 (7)	−0.0008 (7)	0.0035 (7)
C17	0.0285 (9)	0.0263 (9)	0.0234 (8)	0.0040 (7)	−0.0013 (7)	0.0019 (7)
C18	0.0297 (9)	0.0294 (9)	0.0172 (8)	0.0063 (7)	−0.0020 (7)	0.0008 (7)
C19	0.0348 (10)	0.0374 (10)	0.0200 (8)	0.0098 (8)	0.0000 (7)	−0.0036 (8)
C20	0.0311 (10)	0.0230 (8)	0.0325 (9)	0.0077 (7)	−0.0005 (7)	−0.0013 (8)

### Geometric parameters (Å, °)

**Table:**

O1—C7	1.259 (2)	C8—C9	1.393 (2)
O2—C7	1.260 (2)	C8—C13	1.394 (2)
O3—C11	1.341 (2)	C9—C10	1.379 (3)
O3—H3O	0.8401	C9—H9	0.9500
N1—N2	1.263 (2)	C10—C11	1.392 (2)
N1—C2	1.432 (2)	C10—H10	0.9500
N2—C8	1.417 (2)	C11—C12	1.398 (2)
N3—C17	1.351 (2)	C12—C13	1.381 (2)
N3—C16	1.352 (2)	C12—H12	0.9500
N3—H3N	0.8801	C13—H13	0.9500
N4—C14	1.339 (2)	C14—C15	1.421 (2)
N4—C20	1.459 (2)	C14—C18	1.426 (2)
N4—C19	1.465 (2)	C15—C16	1.358 (3)
C1—C2	1.400 (2)	C15—H15	0.9500
C1—C6	1.401 (2)	C16—H16	0.9500
C1—C7	1.513 (2)	C17—C18	1.362 (2)
C2—C3	1.404 (2)	C17—H17	0.9500
C3—C4	1.385 (2)	C18—H18	0.9500
C3—H3	0.9500	C19—H19A	0.9800
C4—C5	1.390 (2)	C19—H19B	0.9800
C4—H4	0.9500	C19—H19C	0.9800
C5—C6	1.390 (2)	C20—H20A	0.9800
C5—H5	0.9500	C20—H20B	0.9800
C6—H6	0.9500	C20—H20C	0.9800
			
C11—O3—H3O	114.6	C11—C10—H10	120.1
N2—N1—C2	111.98 (14)	O3—C11—C10	118.12 (15)
N1—N2—C8	115.30 (14)	O3—C11—C12	122.74 (16)
C17—N3—C16	119.54 (15)	C10—C11—C12	119.15 (15)
C17—N3—H3N	121.3	C13—C12—C11	120.58 (16)
C16—N3—H3N	119.0	C13—C12—H12	119.7
C14—N4—C20	121.47 (15)	C11—C12—H12	119.7
C14—N4—C19	121.06 (15)	C12—C13—C8	120.40 (16)
C20—N4—C19	117.45 (15)	C12—C13—H13	119.8
C2—C1—C6	118.72 (15)	C8—C13—H13	119.8
C2—C1—C7	123.06 (14)	N4—C14—C15	122.72 (16)
C6—C1—C7	118.07 (14)	N4—C14—C18	121.14 (15)
C1—C2—C3	119.81 (15)	C15—C14—C18	116.14 (15)
C1—C2—N1	118.78 (15)	C16—C15—C14	119.77 (16)
C3—C2—N1	121.37 (15)	C16—C15—H15	120.1
C4—C3—C2	120.54 (16)	C14—C15—H15	120.1
C4—C3—H3	119.7	N3—C16—C15	122.57 (16)
C2—C3—H3	119.7	N3—C16—H16	118.7
C3—C4—C5	119.84 (16)	C15—C16—H16	118.7
C3—C4—H4	120.1	N3—C17—C18	121.32 (16)
C5—C4—H4	120.1	N3—C17—H17	119.3
C6—C5—C4	119.85 (16)	C18—C17—H17	119.3
C6—C5—H5	120.1	C17—C18—C14	120.67 (16)
C4—C5—H5	120.1	C17—C18—H18	119.7
C5—C6—C1	121.05 (15)	C14—C18—H18	119.7
C5—C6—H6	119.5	N4—C19—H19A	109.5
C1—C6—H6	119.5	N4—C19—H19B	109.5
O1—C7—O2	124.71 (16)	H19A—C19—H19B	109.5
O1—C7—C1	117.02 (14)	N4—C19—H19C	109.5
O2—C7—C1	118.22 (14)	H19A—C19—H19C	109.5
C9—C8—C13	118.59 (16)	H19B—C19—H19C	109.5
C9—C8—N2	115.54 (15)	N4—C20—H20A	109.5
C13—C8—N2	125.76 (15)	N4—C20—H20B	109.5
C10—C9—C8	121.40 (17)	H20A—C20—H20B	109.5
C10—C9—H9	119.3	N4—C20—H20C	109.5
C8—C9—H9	119.3	H20A—C20—H20C	109.5
C9—C10—C11	119.87 (17)	H20B—C20—H20C	109.5
C9—C10—H10	120.1		
			
C2—N1—N2—C8	179.04 (13)	N2—C8—C9—C10	176.36 (19)
C6—C1—C2—C3	−3.6 (2)	C8—C9—C10—C11	−0.2 (3)
C7—C1—C2—C3	−179.01 (15)	C9—C10—C11—O3	−179.18 (18)
C6—C1—C2—N1	178.65 (14)	C9—C10—C11—C12	0.2 (3)
C7—C1—C2—N1	3.2 (2)	O3—C11—C12—C13	179.40 (17)
N2—N1—C2—C1	−148.88 (15)	C10—C11—C12—C13	0.0 (3)
N2—N1—C2—C3	33.4 (2)	C11—C12—C13—C8	−0.3 (3)
C1—C2—C3—C4	4.2 (2)	C9—C8—C13—C12	0.3 (3)
N1—C2—C3—C4	−178.05 (15)	N2—C8—C13—C12	−175.72 (17)
C2—C3—C4—C5	−1.1 (2)	C20—N4—C14—C15	2.5 (3)
C3—C4—C5—C6	−2.6 (3)	C19—N4—C14—C15	−179.04 (17)
C4—C5—C6—C1	3.2 (3)	C20—N4—C14—C18	−177.34 (17)
C2—C1—C6—C5	−0.1 (2)	C19—N4—C14—C18	1.1 (3)
C7—C1—C6—C5	175.56 (15)	N4—C14—C15—C16	−179.58 (17)
C2—C1—C7—O1	−134.83 (17)	C18—C14—C15—C16	0.3 (2)
C6—C1—C7—O1	49.7 (2)	C17—N3—C16—C15	0.4 (3)
C2—C1—C7—O2	47.4 (2)	C14—C15—C16—N3	−0.3 (3)
C6—C1—C7—O2	−128.09 (17)	C16—N3—C17—C18	−0.6 (3)
N1—N2—C8—C9	−175.62 (16)	N3—C17—C18—C14	0.7 (3)
N1—N2—C8—C13	0.5 (2)	N4—C14—C18—C17	179.35 (17)
C13—C8—C9—C10	−0.1 (3)	C15—C14—C18—C17	−0.5 (3)

### Hydrogen-bond geometry (Å, °)

**Table:**

*D*—H···*A*	*D*—H	H···*A*	*D*···*A*	*D*—H···*A*
O3—H3o···O2^i^	0.84	1.77	2.602 (2)	174
N3—H3n···O1	0.88	1.78	2.641 (2)	166
C15—H15···O2^ii^	0.95	2.41	3.300 (3)	157
C19—H19a···O3^iii^	0.98	2.58	2.968 (3)	103

Symmetry codes: (i) −*x*+1, −*y*+1, −*z*+1; (ii) −*x*+3/2, *y*−1/2, −*z*+1/2; (iii) *x*, *y*, *z*−1.
